# Utilization of glucagon-like peptide-1 receptor agonists in children and adolescents in China: a real-world study

**DOI:** 10.3389/fendo.2023.1170127

**Published:** 2023-06-13

**Authors:** Yilong Yan, Ying Gong, Meizhu Jiang, Yiming Gao, Shanshan Guo, Jiping Huo, Zhigang Zhao, Cao Li

**Affiliations:** ^1^ Department of Pharmacy, Beijing Tiantan Hospital, Capital Medical University, Beijing, China; ^2^ Department of Clinical Pharmacology, School of Pharmaceutical Sciences, Capital Medical University, Beijing, China; ^3^ Department of Pharmacy, Dongfang Hospital, Beijing University of Chinese Medicine, Beijing, China

**Keywords:** GLP-1RA, obesity, diabetes, children, adolescents

## Abstract

**Background:**

Glucagon-like peptide-1 receptor agonists (GLP-1RAs) have been widely used in treating type 2 diabetes mellitus (T2DM) and obesity in adults, but scientific research about the indication in children and adolescents is scarce. The current study aims to explore the prescriptions of GLP-1RAs in children and adolescents in China and to evaluate its rationality.

**Methods:**

GLP-1RA prescriptions of children and adolescents were retrospectively obtained from the Hospital Prescription Analysis Cooperative Project. The study extracted information on patient’s demographic characteristics, monotherapy and combination therapy of GLP-1RAs, and trends in GLP-1RA usage from 2016 to 2021. The rationality of GLP-1RA prescriptions was comprehensively assessed based on the indications approved by China National Medical Products Administration (NMPA), the U.S. Food and Drug Administration (FDA), European Medicines Agency (EMA), Pharmaceuticals and Medical Devices Agency (PMDA), and published randomized controlled trials (RCTs).

**Results:**

A total of 234 prescriptions from 46 hospitals were included, with a median age of 17 years old. The majority of patients were diagnosed with overweight/obesity or prediabetes/diabetes, accounting for 43.59% and 46.15%, respectively. There were 88 patients on GLP-1RA monotherapy. GLP-1RAs plus metformin was the most common combination therapy (38.89%). 12.39% of patients were found a co-administration with orlistat. The share of overweight/obesity prescriptions increased from 27% in 2016 to 54% in 2021, whereas prediabetes/diabetes prescriptions declined from 55% to 42%. The prescriptions were divided into appropriate and questionable groups according to the diagnosis, and the potentially questionable prescription was related to age (*p* = 0.017), department visited (*p* = 0.002), and any hospitalization (*p* < 0.001).

**Conclusions:**

This study described the prescribing of GLP-1RAs in children and adolescents. Our findings indicated that the utilization of GLP-1RAs has increased from 2016 to 2021. There was a strong basis for administering GLP-1RAs in overweight/obesity and prediabetes/diabetes, whereas the evidence was insufficient in other conditions. It is crucial to demand robust and sustained efforts to enhance the awareness of the safety of utilization of GLP-1RAs in children and adolescents.

## 1 Introduction

Glucagon-like peptide-1 (GLP-1) is an endogenous intestinal glucagon secreted by intestinal L-cells. Glucagon-like peptide-1 receptor agonists (GLP-1RAs) exert hypoglycemic effects by activating GLP-1 receptors to stimulate insulin secretion and inhibit glucagon secretion in a glucose concentration-dependent manner, while increasing glucose uptake by muscle and adipose tissue and inhibiting hepatic glucose production. In addition, GLP-1RAs can delay gastric emptying and suppress appetite (1).

Since the approval of the first GLP-1RA exenatide in 2005, more types of this class of drugs have gradually become available. According to their pharmacokinetics, GLP-1RAs marketed in China are classified into short-acting benaglutide, exenatide, and lixisenatide, as well as long-acting liraglutide, exenatide weekly formulations, dulaglutide, and loxenatide (1). To be mentioned, benaglutide and loxenatide are produced and marketed only in China up to now. These drugs have been proven in clinical trials and real-world studies to be effective in controlling blood glucose levels in adults with type 2 diabetes mellitus (T2DM), as well as in weight reduction. Some of these GLP-1RAs may have a beneficial impact on the cardiovascular system through their effects on heart rate, blood pressure, and cardiac hemodynamic response (2).

The prevalence of obesity in children and adolescents has been increasing in recent years, accompanied by a large number of diabetes cases. However, prevention and management of obesity are severely lacking in low- and middle-income countries (LMICs) (3). As the second largest economy and LMICs simultaneously, China faces serious challenges in diabetes and obesity in pediatrics. The estimated prevalence of overweight and obese children aged 6 to 17 from 2015 to 2019 was 11.1% and 7.9% in China, respectively (4). According to Chinese hospital data, glycemic control among children with diabetes has improved over the past decade but still lags behind that in high-income countries (5). Epidemiological research reported that the prevalence of obesity in children with T2DM was 75.27% (6) and the course of T2DM in childhood and adolescence may be faster and more devastating than in patients with later onset of the disease, leading to poor quality of life (7). Today, GLP-1RAs are gradually being used in children and adolescents. However, there is a relative lack of GLP-1RA studies founded on children and adolescents compared with adults, and the rate of adverse effects such as nausea has risen (8), so clinical use should be strictly controlled for indications. Currently, FDA has approved liraglutide for the treatment of T2DM in children and adolescents older than 10 years of age and obesity in adolescents 12-17 years of age; exenatide weekly formulations are available for use in T2DM patients 10-17 years of age; and once-weekly semaglutide is approved for the treatment of obesity in pediatric patients aged 12 years and older. Besides, EMA has approved liraglutide for the treatment of obesity and T2DM in children and adolescents older than 12 years of age; exenatide is available for use in T2DM patients older than 10 years of age. PMDA is not currently approved for use younger than 18 years of age.

The purpose of the study was to analyze (1) the utilization of GLP-1RAs in children and adolescents in China, (2) the rationality of prescribing, and (3) to provide a reference for the clinical rational use of GLP-1RAs.

## 2 Methods

### 2.1 Study design

This study was carried out as a retrospective analysis of prescribing GLP-1RAs in children and adolescents using descriptive statistics. The correctness of diagnosis was completed by trained boarded doctors believed adhering guidelines. For instance, briefly the diagnostic criteria for diabetes is the presence of typical diabetic symptoms plus a plasma glucose level greater than 11.1 mmol/L at any time, or a fasting plasma glucose level greater than 7.0 mmol/L, or a 2-hour plasma glucose level greater than 11.1 mmol/L in an OGTT test (1). Besides, BMI percentile range of 85th to 94th percentile for age and sex is considered overweight, and BMI percentile range at or above 95th percentile for age and sex is considered obesity (9). The original diagnosis in the prescription was coded and classified according to the International Classification of Diseases, 10th Edition (ICD-10). Assess the rationality of applying GLP-1RAs in children and adolescents based on FDA-approved indications. Besides, literature databases search was performed in PubMed, the Cochrane Library, and CNKI from 1 May 2004 to 31 December 2022, attempting to explore more evidence-based applications of GLP-1RA in children and adolescents. Evaluated the quality of evidence, this study defines “appropriate” as having at least one RCT that demonstrates an indication for the effectiveness of any type of GLP-1RAs in children and adolescents, and “questionable” if there is no evidence of this level.

### 2.2 Study sample

The prescriptions were obtained from the database of Hospital Prescription Analysis Cooperative Project, which was conducted by the Chinese Pharmaceutical Association. GLP-1RA prescription was extracted from participating hospitals on ten randomized sampling workdays for each quarter. The study collected patients’ data of age, gender, year, region, department visited, reimbursement, hospital level, any hospitalization, total payment, comorbidities, and medications. Prescription information was acquired from 46 sample hospitals in nine cities or provinces of Beijing, Shenyang, Chengdu, Guangzhou, Harbin, Hangzhou, Shanghai, Tianjin, and Zhengzhou from 2016 to 2021. The present study was approved by the ethics committee at Beijing Tiantan Hospital, Capital Medical University.

Inclusion criteria: (1) the prescription period was from January 1, 2016, to December 31, 2021; (2) the prescription drug contained at least one GLP-1RA; (3) the age of patients was from 1 to 18 years old. Exclusion criteria: (1) repeated prescriptions; (2) prescriptions lacking gender or diagnostic information.

### 2.3 Statistical analysis

In this study, we conducted a descriptive analysis of GLP-1RA prescriptions. The overall trends in GLP-1RA utilization were characterized by the number of prescriptions. Monotherapy and combination therapy were analyzed for children and adolescents with obesity or diabetes. Qualitative variables were expressed as absolute and relative frequencies, while quantitative variables were described by the median, interquartile range (IQR) for the reason that they do not conform to a normal distribution. We classified the prescriptions into appropriate and questionable groups. Non-parametric tests, Chi-square tests, and Fisher exact tests were performed using SPSS (V.26). *P*<0.05 was considered statistically significant.

## 3 Results

### 3.1 Descriptive statistics of total prescriptions

**Figure 1:**
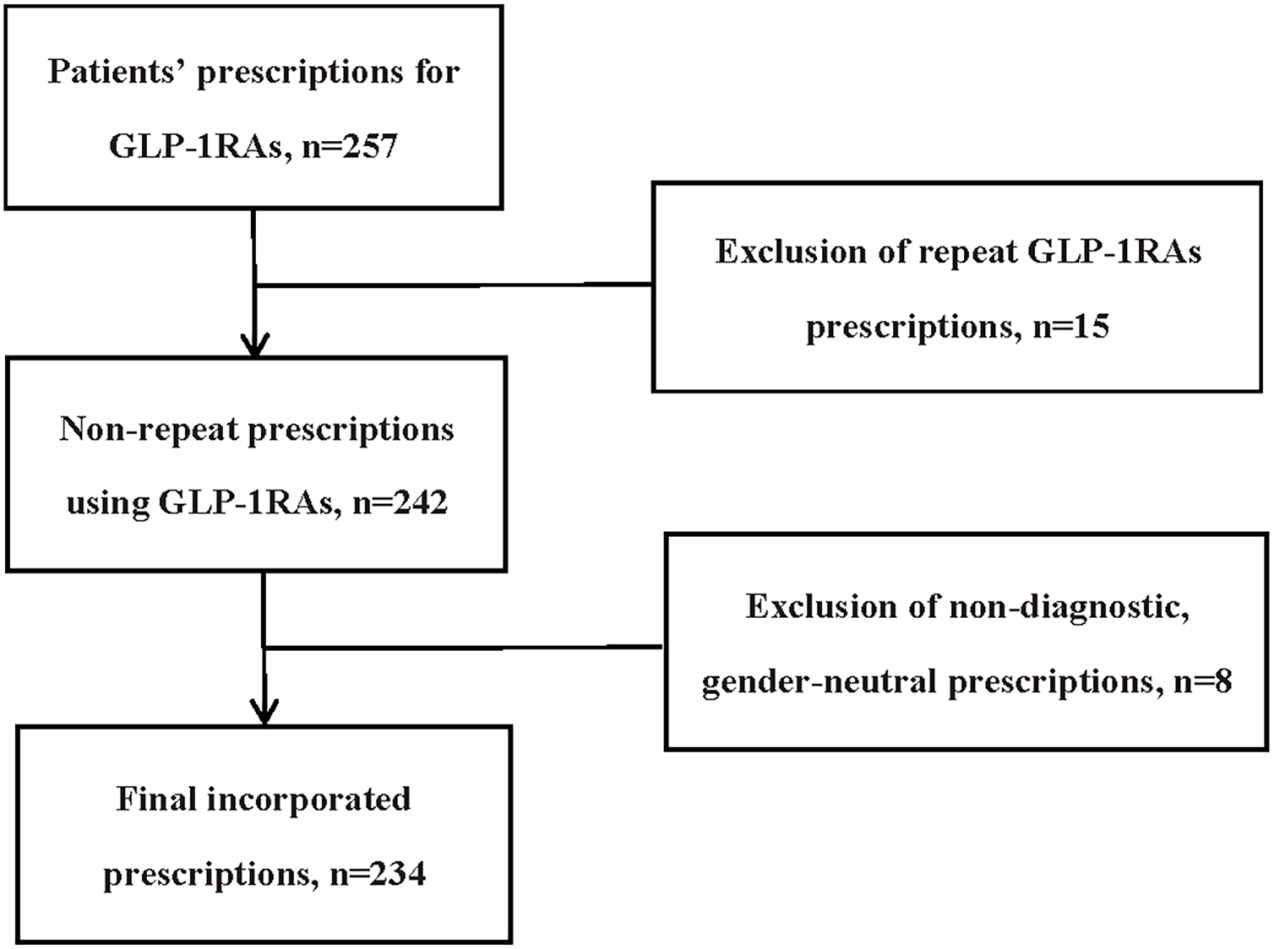
Flow diagram for study cohort inclusion.

The study consisted of 234 children and adolescents with GLP-1RA treatment after applying the inclusion and exclusion criteria (**Figure 1**). From 2016 to 2021, the proportions of GLP-1RA use increased from 9.40% to 34.62%. The median age of the patients was 17 years with a slightly higher percentage of females than males (**Table 1**). A great number of patients were treated in endocrinology departments (80.77%). Most of the prescriptions were acquired from second-tier cities (57.26%) and tertiary hospitals accounted for a large proportion (97.44%). Compared with prescriptions acquired in inpatient facilities, prescriptions acquired in outpatient facilities presented significantly higher proportions (76.5% *vs* 24.5%) (**Table 1**). Nearly half of the patients were not covered by insurance. Thus, the expenses with a median total cost of 678 yuan (99.60 USD 2023. Feb) in each visit are covered by the family. The largest share of diagnoses was prediabetes/diabetes (46.15%), and the number of overweight/obesity followed closely behind (43.59%). However, the percentage of patients with both overweight/obesity and prediabetes/diabetes was much lower than the patients with a single diagnosis of these two diseases (8.97%). GLP-1RA with metformin (MET) was the most prescribed combination therapy of hypoglycemic drugs, reaching 38.89%. In addition, GLP-1RAs were also commonly combined with orlistat (12.39%) (**Table 1**).

**Table 1:** Demographic characteristics of patients.

Characteristic	Patients (N=234)
Age, [median (IQR)]	17 (16-18)
Gender, n (%)	
Male	111 (47.44)
Female	123 (52.56)
Year, n (%)	
2016	22 (9.40)
2017	15 (6.41)
2018	18 (7.69)
2019	34 (14.53)
2020	64 (27.35)
2021	81 (34.62)
Region, n (%)	
First-tier cities	100 (42.74)
Second-tier cities	134 (57.26)
Department Visited, n (%)	
Endocrinology	189 (80.77)
General/internal medicine	17 (7.27)
Pediatrics	6 (2.56)
Others	22 (9.40)
Reimbursement, n (%)	
Full or partial	119 (50.85)
Self-pay	97 (41.45)
N/A	18 (7.70)
Hospital level, n (%)	
Tertiary	228 (97.44)
Secondary	6 (2.56)
Any hospitalization, n (%)	
Inpatient	55 (23.50)
Outpatient	179 (76.50)
Total Payment, [median (IQR)]	678.00 (410.00-1017.00)
Comorbidities, n (%)	
Prediabetes/diabetes	108 (46.15)
Overweight/obesity	102 (43.59)
Hyperlipidemia	26 (11.11)
PCOS	25 (10.68)
Hyperinsulinemia/IR	22 (9.40)
Overweight/obesity+Prediabetes/diabetes	21 (8.97)
Hypertension	20 (8.55)
Hyperuricemia	18 (7.69)
Other metabolic diseases^a^	18 (7.69)
Others	13 (5.56)
Medications, n(%)	
MET	91 (38.89)
Thiazolidinedione	21 (8.97)
Insulin	16 (6.84)
Glycosidase inhibitors	13 (5.56)
Other hypoglycemic drugs^b^	4 (1.71)
Orlistat	29 (12.39)
Antihypertensive	16 (6.84)
Lipid-lowering drugs	13 (5.56)
Uric acid reduction medicine	11 (4.70)
Others	20 (8.55)

Patient characteristics are expressed as number of patients (values between parentheses indicate percentages) or median (IQR).

IR, insulin resistance; IQR, interquartile range; N/A, Not applicable.

^a^Other metabolic diseases include metabolic syndrome, hypothalamus syndrome, Prader-Willi syndrome and thyroid disease.

^b^Other hypoglycemic drugs include dipeptidyl peptidase-4 (DPP4) inhibitors and SGLT2 inhibitors.

### 3.2 Overall trends in the utilization of GLP-1RAs in obese and diabetic patients

**Figure 2:**
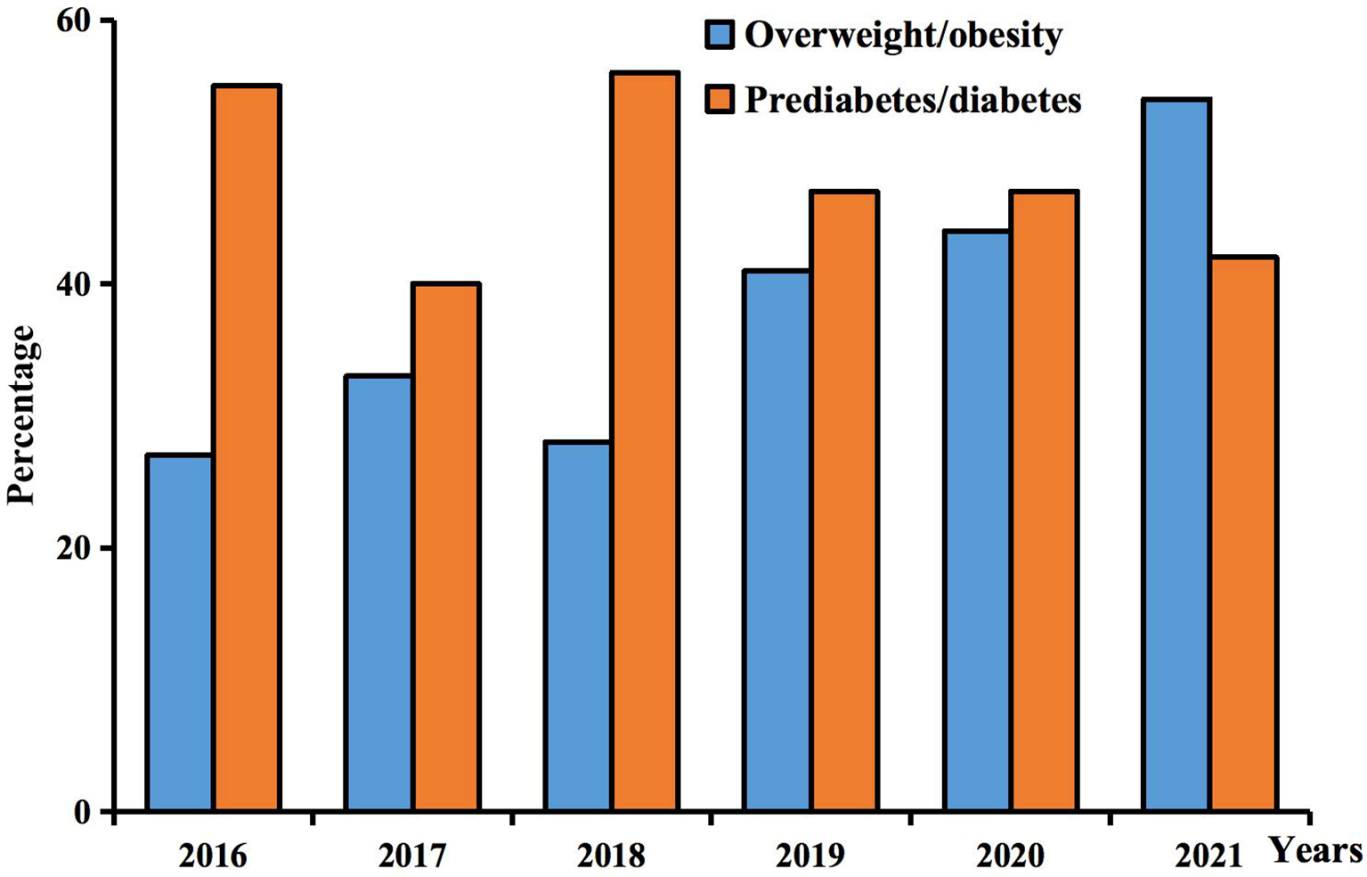
Trends in the use of GLP-1RAs in obese and diabetic patients.

From 2016 to 2021, prediabetes/diabetes accounted for a substantial proportion of our study (≧40%). But throughout the overall trends, the ratio of overweight/obesity increased from 27% in 2016 to 54% in 2021, while prediabetes/diabetes declined from 55% to 42%. The share of prediabetes/diabetes was higher than that of overweight/obesity until 2021, when overweight/obesity surpassed prediabetes/diabetes (**Figure 2**).

### 3.3 Rational analysis of using GLP-1RAs

The 234 prescriptions were divided into appropriate and questionable groups according to the diagnosis based on the evidence. Comparing the baseline characteristics of the two groups, the median age was 17 years in the appropriate group and 16 years in the questionable group, with significant differences in age (*p* = 0.017), department visited (*p* = 0.002), and any hospitalization (*p*<0.001). While gender, year, region, reimbursement, hospital level, and total payment were not significantly different (all *p*>0.05; **Table 2**).

**Table 2:** Comparison of characteristics of appropriate and questionable prescriptions.

Characteristic	Appropriate	Questionable	*P*
Total number, n (%)	189 (80.77)	45 (19.23)	
Age, [median (IQR)]	17 (16-18)	16 (15-17)	0.017*
Gender, n (%)			0.908
Male	90 (38.46)	21 (8.97)	
Female	99 (42.31)	24 (10.26)	
Year, n (%)			0.869
16-18	44 (18.80)	11 (4.70)	
19-21	145 (61.97)	34 (14.53)	
Region, n (%)			0.796
First-tier cities	80 (34.19)	20 (8.55)	
Second-tier cities	109 (46.58)	25 (10.68)	
Department visited, n (%)			0.002**
Endocrinology	155 (66.24)	34 (14.53)	
General/internal medicine	16 (6.83)	1 (0.43)	
Pediatrics	1 (0.43)	5 (2.14)	
Others	17 (7.26)	5 (2.14)	
Reimbursement, n (%)			0.198
Full or partial	100 (42.73)	19 (8.12)	
Self-pay	77 (32.91)	20 (8.55)	
N/A	12 (5.13)	6 (2.56)	
Hospital level, n (%)			1.000
3	184 (78.63)	44 (18.80)	
2	5 (2.14)	1 (0.43)	
Any hospitalization, n (%)			0.000***
Inpatient	33 (14.10)	22 (9.40)	
Outpatient	156 (66.67)	23 (9.83)	
Total payment, [median (IQR)]	678.00 (410.00-1017.00)	678.00 (339.00-853.00)	0.112

Age and total payment were compared using the non-parametric test; categorical values were compared using the chi-square test or fisher exact test: fisher exact test for department visited.

*Statistically significant. *P<0.05; **P<0.01; ***P<0.001.

Of all conditions in which GLP-1RA prescriptions applied, overweight/obesity and prediabetes/diabetes were the most common diagnoses. GLP-1RA monotherapy appeared the most frequently (n=88), with similar proportions of patients diagnosed with overweight/obesity or prediabetes/diabetes. However, the proportion of patients with these two concurrent diseases was only 9.09%. Among the combination therapy, GLP-1RA with MET accounted for the largest amount (n=28), with no less than 50% in the diagnosis of overweight/obesity or prediabetes/diabetes. GLP-1RA with orlistat was mainly used for obese patients (71.43%), as well as three-drug combination therapy of GLP-1RA with MET and orlistat (50.00%). GLP-1RA with MET and other hypoglycemic drugs was popular in prediabetes/diabetes (75.00%). The diagnosis of prediabetes/diabetes was often included when GLP-1RAs were used in two-drug combination therapy with other hypoglycemic drugs (100%) (**Table 3**).

**Table 3:** Combination therapy or monotherapy in overweight/obesity and prediabetes/diabetes.

Combination therapy	n	Overweight/obesity	Prediabetes/diabetes	Both overweight/obesity and prediabetes/diabetes
GLP-1RA monotherapy	88	38 (43.18)	37 (42.05)	8 (9.09)
GLP-1RA+ MET	28	16 (57.14)	14 (50.00)	4 (14.29)
GLP-1RA+ Orlistat	7	5 (71.43)	1 (14.29)	1 (14.29)
GLP-1RA+ MET+ Orlistat	6	3 (50.00)	0 (0.00)	0 (0.00)
GLP-1RA+ MET+ Other hypoglycemic drugs^a^	16	6 (37.50)	12 (75.00)	2 (12.50)
GLP-1RA+ Other hypoglycemic drugs	8	2 (25.00)	8 (100.00)	2 (25.00)

^a^Other hypoglycemic drugs include glycosidase inhibitors, DPP4 inhibitors, SGLT2 inhibitors, insulin and thiazolidinedione.

## 4 Discussion

The research investigated the real-world utilization of GLP-1RAs in children and adolescents in China based on a large database. The included type of GLP-1RAs in the database was exenatide, liraglutide, lixisenatide, benaglutide, dulaglutide, and polyethylene glycol loxenatide. Our present study demonstrated a steady increase in the frequency of GLP-1RA use from 2016 to 2021. Such a trend might be related to the time of GLP-1RAs covered under medical insurance and indication approval for children and adolescents. For instance, as the most frequently prescribed type in this study, liraglutide entered the Medicare list in 2017, followed by other types of GLP-1RAs in succession, which might lead to an increase in drug prescription.

The demographic characteristics of this study showed that GLP-1RA utilization was primarily observed in 16 to 18 years old, possibly due to the soaring prevalence of diabetes and obesity among adolescents in recent years. Over the past 30 years, T2DM has a shifting gradually in adult disorders and pediatric diseases (10). At the same time, the World Obesity Federation estimated that 206 million children and adolescents aged 5-19 would be obese by 2025 (11). There were slightly more females than males in all prescriptions. This result could possibly be explained by the fact that females faced menarche during their adolescence, which might subsequently develop more endocrine and metabolism problems. As a common endocrine disorder, the global prevalence of PCOS among adolescent girls ranges from 1.14% to 11.04%. A meta-analysis reported that about one-fifth of girls with T2DM were accompanied by PCOS (10). In all prescriptions, the interquartile range of total payment varied from 410 to 1017 yuan (60.06 to 148.99 USD 2023. Feb), with 50.85% of patients being reimbursed for certain expenses. It was considered that pharmacoeconomics was an important factor affecting the selection of GLP-1RAs. Recent research described that the use of GLP-1RAs increased significantly under Medicaid expansion (12).

In our study, a large proportion of children and adolescents suffered from overweight/obesity, accounting for 43.59%. The ratio of patients with overweight/obesity increased from 27% in 2016 to 54% in 2021. As the prevalence increases drastically in recent decades, it is of great importance that evidenced weight management should be adapted for children and adolescents (13).The relationship between GLP-1RA application and weight loss during adolescence has been investigated in increasing amounts. In a randomized double-blind trial conducted on obese adolescents, 3.0mg liraglutide was proven to have a greater impact on body mass index (BMI) reduction compared with placebo (14). The results of previous research demonstrated that exenatide reduced BMI by 1.7 kg/m^2^ in extremely obese children and adolescents aged 9-16 years (15). As a newly approved GLP-1RA, trials of semaglutide treatment with a dose of 2.4mg once-weekly in adolescents have indicated the mean change in BMI from baseline to week 68 was -16.1% (16), which provides strong evidence for its effectiveness in pediatric obesity.

T1DM or T2DM are classified as “diabetes” without distinction in our research. Prediabetes includes impaired fasting glucose (IFG) and impaired glucose tolerance (IGT) (1). 46.15% of the prescriptions contained a diagnosis of prediabetes/diabetes, which was the most frequent diagnosis in the study. The overall trends of prediabetes/diabetes declined from 55% in 2016 to 42% in 2021. A large cross-sectional study of 6365 adolescents showed an overall prevalence of 0.9%. Various characteristics of adolescents are associated with the diagnosis of diabetes, such as gender, marital status of parents, smoking, or use of illegal drugs (17), which may lead to a high prevalence of diabetes in adolescents. Liraglutide and exenatide were approved for T2DM in children and adolescents. In a study of T2DM patients aged 10-17 years, mean HbA1c levels decreased by 0.64 percentage points in the 1.8 mg liraglutide group, while increasing by 0.42 percentage points in the placebo group (18). Once-a-week exenatide resulted in a significant decrease in HbA1c at 24 weeks in adolescents with T2DM and was well tolerated (19). Among all patients with prediabetes/diabetes, 71.29% were not classified (**Supplementary Table 1**). Only one patient was accurately diagnosed with T1DM and treated with insulin. Insulin is the recommended medication for T1DM in children and adolescents. Epidemiological studies have shown that children with T1DM continue to have difficulty accessing insulin preparations in LMICs (20). In our study, there were only 16 patients received insulin in combination, and more than half of them did not specify the type of diabetes. Guidelines suggest that insulin is initially required for T2DM with ketoacidosis or HbA1c ≥ 8.5% in children and adolescents (21). Due to the absence of laboratory indicators and clear diagnosis, we speculated that the low rate of insulin use in our study might be explained by the small number of patients with T1DM and the mild disease of T2DM.

There was a large range of complications in our study, among which hyperlipidemia accounted for 11.11% and hypertension accounted for 8.55%. Both T2DM and obesity are closely associated with the occurrence of cardiovascular disease (CVD). GLP-1RA has been identified as an additional method to lower lipid and blood pressure levels, thereby reducing the risk of atherosclerosis (22). Previous evidence suggested that obesity in children and adolescents was associated with unhealthy levels of blood fat, insulin, and blood pressure. Compared with people of the same age and gender, children and adolescents with higher BMI levels may have multiple cardiovascular risk factors (23). It has been demonstrated that different types of GLP-1RAs have individual differences in efficacy and vascular protection (24). For instance, liraglutide has been proven to lower the risk of major adverse cardiovascular events (MACE), while lixisenatide and slow-release exenatide had a neutral effect (25).

There were 88 prescriptions for GLP-1RA monotherapy, among which 43.18% were diagnosed with overweight/obesity and 42.05% were diagnosed with prediabetes/diabetes. Despite the effect of GLP-1RAs on weight reduction, the indication has not yet been approved in China. It is controversial that obese patients use GLP-1RAs at a greater rate than diabetics. Children and adolescents with both overweight/obesity and prediabetes/diabetes only accounted for 9.09% when GLP-1RA monotherapy was performed. The results indicated that GLP-1RA monotherapy was more likely to be selected for treatment when only one diagnosis of overweight/obesity or prediabetes/diabetes was presented. However, eight patients were using GLP-1RA monotherapy among twenty-one patients suffering from both overweight/obesity and prediabetes/diabetes, which represented a significant percentage.

In our research, 28 (11.97%) patients were treated with a two-drug combination of GLP-1RA and MET. Compared with MET alone, exenatide combined with MET not only has a greater glycemic control effect, but also reduces inflammation and protects β-cells (26). On the basis of MET treatment, liraglutide in T2DM patients has a superior effect on blood glucose control, weight reduction, and lowering the incidence of hypoglycemia (27). It has been shown that the combination therapy of GLP-1RA and MET has a gender difference, such as exenatide combined with MET is better in female patients than in males (28). Moreover, studies in obese women with gestational diabetes indicated that liraglutide combined with MET reduced triglyceride and triglyceride/high-density lipoprotein (HDL) cholesterol ratio compared with MET monotherapy (29). Complications should be considered when combined with other hypoglycemic drugs. For instance, GLP-1RAs or sodium-glucose cotransporter-2 (SGLT2) inhibitors have atherosclerotic cardiovascular disease (ASCVD) benefits. It is recommended to combine with MET in patients with T2DM at high cardiovascular risk, as long as there are no contraindications (1). In this study, there were 16 prescriptions of GLP-1RA with MET and other hypoglycemic drugs, and 8 prescriptions were only used in combination with other hypoglycemic drugs, among which the popular combination was GLP-1RA combined with SGLT2 inhibitors. The combination of exenatide and dapagliflozin improves blood glucose indexes and cardiovascular risk factors in patients with T2DM who are poorly controlled by MET monotherapy (30). However, the International Society for Pediatric and Adolescent Diabetes (ISPAD) recommends that initial treatment in pediatric T2DM patients choose MET and/or insulin alone or in combination (21). If patients do not have contraindications for MET, this combination does not meet the guideline’s recommendation. In this study, the combination therapy included 12.39% of the prescriptions for GLP-1RA and orlistat. Among this two-drug combination, 71.43% of the patients were diagnosed with obesity. These results suggested that GLP-1RA and orlistat are regular ways of obesity treatment. It’s worth noting that Clinical Practice Guideline advocates intensive health behavior and lifestyle treatment for childhood obesity, and for children younger than 12 years of age, there is insufficient evidence to provide indications for the use of medication (31). Orlistat is a commonly used weight-loss drug in China. As a gastrointestinal lipase inhibitor, orlistat can reduce the absorption and utilization of fat by the intestinal mucosa. In 2003, orlistat was approved by the FDA for use in adolescents aged 12 to 16 with obesity. A randomized double-blind trial showed that 120mg orlistat can reduce the BMI of obese adolescents aged 12-16 by 0.55kg/m^2^ (32). Orlistat may increase postprandial GLP-1 levels, thereby enhancing insulin response to food, promoting insulin secretion, and slowing postprandial glucose increases in people with T2DM. At the same time, the increase in GLP-1 level will lead to a decrease in food intake, which may lead to weight loss (33). Nevertheless, it is necessary to draw attention to the combination therapy of GLP-1RAs in children and adolescents. They are more prone to adverse reactions when combined with drugs because of differences in absorption, distribution, metabolism, and excretion processes compared to adults, which in turn lead to different drug reactions in children and adolescents (34).

The diagnosis of overweight/obesity and prediabetes/diabetes could be classified as appropriate and the remaining diagnosis is questionable according to the criteria of the two groups. There were 189 prescriptions in the appropriate group, which covered the vast majority of patients with GLP-1RAs. The questionable group contained many kinds of diagnoses, most of which were irrational drug use without robust evidence. The characteristic between the appropriate group and the questionable group may cause a significant difference. The mean age of the appropriate group was 17 years, and in the questionable group was 16 years. The ratio of questionable prescriptions in the departments of pediatrics was significantly higher than that in endocrinology or other departments, which might be due to the variety of diseases covered by pediatrics, consequently, there were more irrational diagnoses for GLP-1RAs. More inpatient comorbidities resulted in a higher percentage of questionable prescriptions than outpatients.

Scientific research for children and adolescents in the questionable group was still far from well-characterized. However, we divided these potentially plausible diagnoses into two parts based on the evidence. The validity of some diagnoses have been verified by RCTs in adults, which may be closely related to obesity or diabetes, and the utilization of GLP-1RAs as an adjuvant medication might have a certain effect. In our study, PCOS patients accounted for 10.68%, while hyperinsulinemia or insulin resistance (IR) accounted for 9.40%. Nearly half of the PCOS patients were obese, and 16% were hyperinsulinemia or IR. PCOS is a highly prevalent disease among women of childbearing age, resulting in endocrine and metabolic disorders (35). Obesity-induced IR and secondary hyperinsulinemia are independent factors affecting PCOS. Irregular menstruation and excessive androgen are the main characteristics of adolescent PCOS (36). The guidelines show that MET is widely used in patients with PCOS to improve IR. In recent years, with the deepening of research, GLP-1RAs have been gradually applied in patients with PCOS, especially those accompanied by obesity. Liraglutide 3 mg once daily can reduce weight and free androgen index in obese women with PCOS (37). Compared with MET monotherapy, exenatide monotherapy or in combination with MET achieve a higher rate of diabetes remission in PCOS patients by increasing insulin secretion after meals (38). At the same time, combination therapy is superior in improving the menstrual cycle, ovulation rate, and free androgen index (39). A recent meta-analysis indicated that liraglutide combined with MET was more effective than MET monotherapy in improving PCOS (40). Although a considerable amount of research has observed that the combination of GLP-1RA and MET has remarkable advantages in improving the menstrual cycle and hormone levels of PCOS patients, there is no evidence of effectiveness when applied to children and adolescents. In addition, both of them are off-label in PCOS with insufficient clinical evidence.

Eleven patients with steatohepatitis were treated with GLP-1RAs, but the specific types of which are not defined in this database. Previous studies have only reported the effectiveness of the treatment of nonalcoholic steatohepatitis (NASH). Recently investigators have examined that semaglutide increased the remission rate of NASH but had no significant effect on the fibrosis stage (41), while liraglutide led to histological improvement in NASH and was well tolerated (42).

Different types of metabolic diseases were contained in this study, such as thyroid disorders and metabolic syndrome. In our study, other metabolic diseases accounted for 7.69%. There was a high prevalence of thyroid disease in patients with GLP-1RAs, including hypothyroidism, hyperthyroidism, and Hashimoto’s thyroiditis. Hypothyroidism patients comprised the largest proportions. Some authors have speculated that subclinical hypothyroidism can increase GLP-1 levels (43). So far, the relationship between GLP-1RAs and thyroid diseases remains controversial. Recent studies have indicated that GLP-1RAs had no effect on the risk of thyroid disease (44), but on the other hand, GLP-1RAs could increase the risk of thyroid cancer and medullary thyroid cancer (45). There were six patients with metabolic syndrome using GLP-1RAs in our research. The prevalence of metabolic syndrome in children is 2.6% in LMICs (46). Metabolic syndrome forms a range of metabolic disorders, including central obesity, insulin resistance, atherogenic dyslipidemia, and hypertension (47). Obesity and IR are regarded as the core of the majority of metabolic syndrome (48). Based on the evidence available, the recommendation for first-line medication is limited to individual treatments for hypertension, hyperglycemia, and hypertriglyceridemia (49). GLP-1RAs are proposed for glucose intolerance and liraglutide for losing weight in order to reduce waist circumference (50).

The other part of the questionable group had weak evidence for the diagnosis (**Supplementary Table 2**), most of which may have diabetic or obese features, but direct evidence of RCTs is still lacking for this section. Acanthosis nigricans (AN) is a common skin condition in which patients are usually obese and may have a history or family history of diabetes or PCOS (51). There is no evidence related to the application of GLP-1RAs. The treatment of AN varies according to the cause of the disease. Two of the four AN patients in our study had combined with obesity. Treating obesity may contribute to the improvement of AN symptoms. However, it is necessary to draw attention that further endocrine evaluation may be required and the presence of malignancy needs to be excluded in patients diagnosed with AN. The utilization of GLP-1RAs, in this case, is probably not reasonable. The genetic disorder Prader-Willi syndrome (PWS) is characterized by severe morbid obesity, often associated with T2DM and uncontrolled taking food (52). Recent evidence suggested that GLP-1RAs were effective in reducing appetite in patients with PWS (53), with potential benefits for weight and blood glucose (54). However, there was still a lack of large-scale clinical trials in children and adolescents in this area.

There were some limitations in our study. A total of 234 prescriptions were included, which was not a large sample size. It was difficult to comprehensively assess the utilization of GLP-1RAs in children and adolescents in China. Studies based on children and adolescents were insufficient, and only literature in Chinese or English was retrieved. In addition, there was a lack of standardization of diagnosis in the database. For example, some diabetes mellitus was not classified into T1DM or T2DM, leading to high rationality of GLP-1RA use. Moreover, the database did not track the treatment effects and adverse reactions, so the effectiveness and safety of patients administering GLP-1RAs could not be determined. Finally, the criterion for evaluating the rationality of unapproved diagnosis was to search for an RCT proving the validity of any kind of GLP-1RAs, but the method could not demonstrate the effectiveness of other kinds of GLP-1RAs. Therefore, some indications for the use of GLP-1RAs might still lack rationality.

## 5 Conclusions

This study evaluated the prescription of GLP-1RAs in children and adolescents in China. GLP-1RAs have multiple effects such as hypoglycemia, weight loss, and vascular protection. However, the existing evidence for children and adolescents is insufficient, so it is necessary to highlight the need for conducting an in-depth and comprehensive evaluation of its safety and effectiveness during adolescence. It should also be noted that there may be differences in the efficacy of different GLP-1RAs, and the type with more robust evidence for the diagnosis should be selected for clinical application whenever possible. In addition, it was found in our research that some indications of GLP-1RA application lacked rationality. Clinical benefits and medication risks of GLP-1RA utilization during adolescence requires further clarification to make correct decisions and ensure medication safety for children and adolescents.

## Data availability statement

The original contributions presented in the study are included in the article/**Supplementary Material**. Further inquiries can be directed to the corresponding authors.

## Author contributions

CL, YGo and ZZ conceived and designed the study. YY, YGo and CL wrote the manuscript. All authors contributed to the article and approved the submitted version.

## References

[1] Guidelines for the prevention and treatment of type 2 diabetes mellitus in China (2020 edition). Chin J Pract Internal Med (2021) 41(08):668–95. doi: 10.19538/j.nk2021080106

[2] Brown E Cuthbertson DJ Wilding JP. Newer GLP-1 receptor agonists and obesity-diabetes. Peptides (2018) 100:61–7. doi: 10.1016/j.peptides.2017.12.009 29412833

[3] Salam RA Padhani ZA Das JK Shaikh AY Hoodbhoy Z Jeelani SM. Effects of lifestyle modification interventions to prevent and manage child and adolescent obesity: a systematic review and meta-analysis. Nutrients (2020) 12(8):2208. doi: 10.3390/nu12082208 PMC7468898 32722112

[4] Pan XF Wang L Pan A. Epidemiology and determinants of obesity in China. Lancet Diabetes Endocrinol (2021) 9(6):373–92. doi: 10.1016/S2213-8587(21)00045-0 34022156

[5] Chen X Pei Z Zhang M Xu Z Zhao Z Lu W. Glycated hemoglobin (HbA1c) concentrations among children and adolescents with diabetes in middle- and low-income countries, 2010-2019: a retrospective chart review and systematic review of literature. Front Endocrinol (2021) 12:651589. doi: 10.3389/fendo.2021.651589 PMC8072468 33912137

[6] Cioana M Deng J Nadarajah A Hou M Qiu Y Chen SSJ. The prevalence of obesity among children with type 2 diabetes: a systematic review and meta-analysis. JAMA Netw Open (2022) 5(12):e2247186. doi: 10.1001/jamanetworkopen.2022.47186 36520430 PMC9856349

[7] Lascar N Brown J Pattison H Barnett AH Bailey CJ Bellary S. Type 2 diabetes in adolescents and young adults. Lancet Diabetes Endocrinol (2018) 6(1):69–80. doi: 10.1016/S2213-8587(17)30186-9 28847479

[8] Ryan PM Seltzer S Hayward NE Rodriguez DA Sless RT Hawkes CP. Safety and efficacy of glucagon-like peptide-1 receptor agonists in children and adolescents with obesity: a meta-analysis. J Pediatr (2021) 236:137–147.e113. doi: 10.1016/j.jpeds.2021.05.009 33984333

[9] Skinner AC Skelton JA. Prevalence and trends in obesity and severe obesity among children in the united states, 1999-2012. JAMA Pediatr (2014) 168(6):561–6. doi: 10.1001/jamapediatrics.2014.21 24710576

[10] Cioana M Deng J Nadarajah A Hou M Qiu Y Chen SSJ. Prevalence of polycystic ovary syndrome in patients with pediatric type 2 diabetes: a systematic review and meta-analysis. JAMA Netw Open (2022) 5(2):e2147454. doi: 10.1001/jamanetworkopen.2021.47454 35166782 PMC8848210

[11] Jebeile H Kelly AS O'Malley G Baur LA. Obesity in children and adolescents: epidemiology, causes, assessment, and management. Lancet Diabetes Endocrinol (2022) 10(5):351–65. doi: 10.1016/S2213-8587(22)00047-X PMC9831747 35248172

[12] Sumarsono A Buckley LF Machado SR Wadhera RK Warraich HJ Desai RJ. Medicaid Expansion and utilization of antihyperglycemic therapies. Diabetes Care (2020) 43(11):2684–90. doi: 10.2337/dc20-0735 PMC8051258 32887711

[13] Lipton-Inga M Manzanarez B Vidmar AP Garcia S Fink C Iverson E. Kids n fitness junior: outcomes of an evidence-based adapted weight management program for children ages three-seven years. Child Obes (2022) 18(1):56–66. doi: 10.1089/chi.2021.0090 34388029 PMC10494906

[14] Kelly AS Auerbach P Barrientos-Perez M Gies I Hale PM Marcus C. A randomized, controlled trial of liraglutide for adolescents with obesity. N Engl J Med (2020) 382(22):2117–28. doi: 10.1056/NEJMoa1916038 32233338

[15] Kelly AS Metzig AM Rudser KD Fitch AK Fox CK Nathan BM. Exenatide as a weight-loss therapy in extreme pediatric obesity: a randomized, controlled pilot study. Obesity (2012) 20(2):364–70. doi: 10.1038/oby.2011.337 PMC3684414 22076596

[16] Weghuber D Barrett T Barrientos-Pérez M Gies I Hesse D Jeppesen OK. Once-weekly semaglutide in adolescents with obesity. N Engl J Med (2022) 387(24):2245–57. doi: 10.1056/NEJMoa2208601 PMC9997064 36322838

[17] Barakat C Yousufzai SJ Booth A Benova L. Prevalence of and risk factors for diabetes mellitus in the school-attending adolescent population of the united Arab Emirates: a large cross-sectional study. BMJ Open (2021) 11(9):e046956. doi: 10.1136/bmjopen-2020-046956 PMC8444241 34526335

[18] Tamborlane WV Barrientos-Pérez M Fainberg U Frimer-Larsen H Hafez M Hale PM. Liraglutide in children and adolescents with type 2 diabetes. N Engl J Med (2019) 381(7):637–46. doi: 10.1056/NEJMoa1903822 31034184

[19] Tamborlane WV Bishai R Geller D Shehadeh N Al-Abdulrazzaq D Vazquez EM. Once-weekly exenatide in youth with type 2 diabetes. Diabetes Care (2022) 45(8):1833–40. doi: 10.2337/dc21-2275 PMC9346995 35679098

[20] Bhutta ZA Salam RA Gomber A Lewis-Watts L Narang T Mbanya JC. A century past the discovery of insulin: global progress and challenges for type 1 diabetes among children and adolescents in low-income and middle-income countries. Lancet (2021) 398(10313):1837–50. doi: 10.1016/S0140-6736(21)02247-9 34774146

[21] Shah AS Zeitler PS Wong J Pena AS Wicklow B Arslanian S. ISPAD clinical practice consensus guidelines 2022: type 2 diabetes in children and adolescents. Pediatr Diabetes (2022) 23(7):872–902. doi: 10.1111/pedi.13409 36161685

[22] Ma X Liu Z Ilyas I Little PJ Kamato D Sahebka A. GLP-1 receptor agonists (GLP-1RAs): cardiovascular actions and therapeutic potential. Int J Biol Sci (2021) 17(8):2050–68. doi: 10.7150/ijbs.59965 PMC8193264 34131405

[23] Freedman DS Mei Z Srinivasan SR Berenson GS Dietz WH. Cardiovascular risk factors and excess adiposity among overweight children and adolescents: the bogalusa heart study. J Pediatr (2007) 150(1):12–17.e12. doi: 10.1016/j.jpeds.2006.08.042 17188605

[24] Iqbal AM Imamudeen N Basheer A Menon S Mohan G Sani TN. Efficacy and cardiovascular safety of GLP-1 receptor analogues. Curr Drug Saf (2021) 16(2):197–206. doi: 10.2174/1574886315999201208212356 33292155

[25] Andrikou E Tsioufis C Andrikou I Leontsinis I Tousoulis D Papanas N. GLP-1 receptor agonists and cardiovascular outcome trials: an update. Hellenic J Cardiol (2019) 60(6):347–51. doi: 10.1016/j.hjc.2018.11.008 30528435

[26] Derosa G Franzetti IG Querci F Carbone A Ciccarelli L Piccinni MN. Exenatide plus metformin compared with metformin alone on β-cell function in patients with type 2 diabetes. Diabetes Med (2012) 29(12):1515–23. doi: 10.1111/j.1464-5491.2012.03699.x 22540883

[27] Nauck M Frid A Hermansen K Shah NS Tankova T Mitha IH. Efficacy and safety comparison of liraglutide, glimepiride, and placebo, all in combination with metformin, in type 2 diabetes: the LEAD (liraglutide effect and action in diabetes)-2 study. Diabetes Care (2009) 32(1):84–90. doi: 10.2337/dc08-1355 18931095 PMC2606836

[28] Quan H Zhang H Wei W Fang T. Gender-related different effects of a combined therapy of exenatide and metformin on overweight or obesity patients with type 2 diabetes mellitus. J Diabetes Complications (2016) 30(4):686–92. doi: 10.1016/j.jdiacomp.2016.01.013 26873871

[29] Elkind-Hirsch KE Shaler D Harris R. Postpartum treatment with liraglutide in combination with metformin versus metformin monotherapy to improve metabolic status and reduce body weight in overweight/obese women with recent gestational diabetes: a double-blind, randomized, placebo-controlled study. J Diabetes Complications (2020) 34(4):107548. doi: 10.1016/j.jdiacomp.2020.107548 32046931

[30] Frías JP Guja C Hardy E Ahmed A Dong F Öhman P. Exenatide once weekly plus dapagliflozin once daily versus exenatide or dapagliflozin alone in patients with type 2 diabetes inadequately controlled with metformin monotherapy (DURATION-8): a 28 week, multicentre, double-blind, phase 3, randomised controlled trial. Lancet Diabetes Endocrinol (2016) 4(12):1004–16. doi: 10.1016/S2213-8587(16)30267-4 27651331

[31] Hampl SE Hassink SG Skinner AC Armstrong SC Barlow SE Bolling CF. Clinical practice guideline for the evaluation and treatment of children and adolescents with obesity. Pediatrics (2023) 151(2):e2022060640. doi: 10.1542/peds.2022-060640 36622115

[32] Chanoine JP Hampl S Jensen C Boldrin M Hauptman J. Effect of orlistat on weight and body composition in obese adolescents: a randomized controlled trial. Jama (2005) 293(23):2873–83. doi: 10.1001/jama.293.23.2873 15956632

[33] Damci T Yalin S Balci H Osar Z Korugan U Ozyazar M. Orlistat augments postprandial increases in glucagon-like peptide 1 in obese type 2 diabetic patients. Diabetes Care (2004) 27(5):1077–80. doi: 10.2337/diacare.27.5.1077 15111524

[34] Morales-Ríos O Jasso-Gutiérrez L Reyes-López A Garduño-Espinosa J Muñoz-Hernández O. Potential drug-drug interactions and their risk factors in pediatric patients admitted to the emergency department of a tertiary care hospital in Mexico. PloS One (2018) 13(1):e0190882. doi: 10.1371/journal.pone.0190882 29304072 PMC5755936

[35] Meier RK. Polycystic ovary syndrome. Nurs Clin North Am (2018) 53(3):407–20. doi: 10.1016/j.cnur.2018.04.008 30100006

[36] Dabadghao P. Polycystic ovary syndrome in adolescents. Best Pract Res Clin Endocrinol Metab (2019) 33(3):101272. doi: 10.1016/j.beem.2019.04.006 31027973

[37] Elkind-Hirsch KE Chappell N Shaler D Storment J Bellanger D. Liraglutide 3 mg on weight, body composition, and hormonal and metabolic parameters in women with obesity and polycystic ovary syndrome: a randomized placebo-controlled-phase 3 study. Fertil Steril (2022) 118(2):371–81. doi: 10.1016/j.fertnstert.2022.04.027 35710599

[38] Tao T Zhang Y Zhu YC Fu JR Wang YY Cai J. Exenatide, metformin, or both for prediabetes in PCOS: a randomized, open-label, parallel-group controlled study. J Clin Endocrinol Metab (2021) 106(3):e1420–32. doi: 10.1210/clinem/dgaa692 PMC8244122 32995892

[39] Elkind-Hirsch K Marrioneaux O Bhushan M Vernor D Bhushan R. Comparison of single and combined treatment with exenatide and metformin on menstrual cyclicity in overweight women with polycystic ovary syndrome. J Clin Endocrinol Metab (2008) 93(7):2670–8. doi: 10.1210/jc.2008-0115 18460557

[40] Ge JJ Wang DJ Song W Song SM Ge WH. The effectiveness and safety of liraglutide in treating overweight/obese patients with polycystic ovary syndrome: a meta-analysis. J Endocrinol Invest (2022) 45(2):261–73. doi: 10.1007/s40618-021-01666-6 34455568

[41] Newsome PN Buchholtz K Cusi K Linder M Okanoue T Ratziu V. A placebo-controlled trial of subcutaneous semaglutide in nonalcoholic steatohepatitis. N Engl J Med (2021) 384(12):1113–24. doi: 10.1056/NEJMoa2028395 33185364

[42] Armstrong MJ Gaunt P Aithal GP Barton D Hull D Parker R. Liraglutide safety and efficacy in patients with non-alcoholic steatohepatitis (LEAN): a multicentre, double-blind, randomised, placebo-controlled phase 2 study. Lancet (2016) 387(10019):679–90. doi: 10.1016/S0140-6736(15)00803-X 26608256

[43] Jin Y Liu H Ma SG Cheng JP Zhang K. Serum levels of glucagon-like peptide (GLP)-1 and GLP-2 in patients with hashimoto's thyroiditis. J Res Med Sci (2015) 20(2):174–7. PMC4400714 25983772

[44] Hu W Song R Cheng R Liu C Guo R Tang W. Use of GLP-1 receptor agonists and occurrence of thyroid disorders: a meta-analysis of randomized controlled trials. Front Endocrinol (2022) 13:927859. doi: 10.3389/fendo.2022.927859 PMC9309474 35898463

[45] Bezin J Gouverneur A Pénichon M Mathieu C Garrel R Hillaire-Buys D. GLP-1 receptor agonists and the risk of thyroid cancer. Diabetes Care (2022) 46(2):384–390. doi: 10.2337/figshare.21357237 36356111

[46] Noubiap JJ Nansseu JR Lontchi-Yimagou E Nkeck JR Nyaga UF Ngouo AT. Global, regional, and country estimates of metabolic syndrome burden in children and adolescents in 2020: a systematic review and modelling analysis. Lancet Child Adolesc Health (2022) 6(3):158–70. doi: 10.1016/S2352-4642(21)00374-6 35051409

[47] Fahed G Aoun L Bou Zerdan M Allam S Bou Zerdan M Bouferraa Y. Metabolic syndrome: updates on pathophysiology and management in 2021. Int J Mol Sci (2022) 23(2):786. doi: 10.3390/ijms23020786 PMC8775991 35054972

[48] Samson SL Garber AJ. Metabolic syndrome. Endocrinol Metab Clin North Am (2014) 43(1):1–23. doi: 10.1016/j.ecl.2013.09.009 24582089

[49] McCracken E Monaghan M Sreenivasan S. Pathophysiology of the metabolic syndrome. Clin Dermatol (2018) 36(1):14–20. doi: 10.1016/j.clindermatol.2017.09.004 29241747

[50] Rask Larsen J Dima L Correll CU Manu P. The pharmacological management of metabolic syndrome. Expert Rev Clin Pharmacol (2018) 11(4):397–410. doi: 10.1080/17512433.2018.1429910 29345505

[51] Patel NU Roach C Alinia H Huang WW Feldman SR. Current treatment options for acanthosis nigricans. Clin Cosmet Investig Dermatol (2018) 11:407–13. doi: 10.2147/CCID.S137527 PMC6086114 30122971

[52] Senda M Ogawa S Nako K Okamura M Sakamoto T Ito S. The glucagon-like peptide-1 analog liraglutide suppresses ghrelin and controls diabetes in a patient with prader-willi syndrome. Endocr J (2012) 59(10):889–94. doi: 10.1507/endocrj.EJ12-0074 22785236

[53] Salehi P Hsu I Azen CG Mittelman SD Geffner ME Jeandron D. Effects of exenatide on weight and appetite in overweight adolescents and young adults with prader-willi syndrome. Pediatr Obes (2017) 12(3):221–8. doi: 10.1111/ijpo.12131 PMC5288290 27071367

[54] Ng NBH Low YW Rajgor DD Low JM Lim YY Loke KY. The effects of glucagon-like peptide (GLP)-1 receptor agonists on weight and glycaemic control in prader-willi syndrome: a systematic review. Clin Endocrinol (2022) 96(2):144–54. doi: 10.1111/cen.14583 34448208

