# Supplementary material for: Utilization of glucagon-like peptide-1 receptor agonists in children and adolescents in China: a real-world study
Source: Front Endocrinol (Lausanne). 2023 Jun 13;14:1170127. doi: 10.3389/fendo.2023.1170127 (PMC10293789; doi:10.3389/fendo.2023.1170127)
Supplement: Supplementary file 1 [file Table_1.docx]

**Supplementary Table 1 MET and insulin use in patients with different types of prediabetes/diabetes**

|  | **Total, n (%)** | **MET, n (%)** | **Insulin, n (%)** |
| --- | --- | --- | --- |
| **Prediabetes** | 5 (4.63) | 0 (0.00) | 0 (0.00) |
| **T1DM** | 1 (0.93) | 0 (0.00) | 1 (0.93) |
| **T2DM** | 25 (23.15) | 10 (9.26) | 6 (5.56) |
| **Diabetes (unclassified)** | 77 (71.29) | 32 (29.63) | 9 (8.33) |
