# Supplementary material for: Utilization of glucagon-like peptide-1 receptor agonists in children and adolescents in China: a real-world study
Source: Front Endocrinol (Lausanne). 2023 Jun 13;14:1170127. doi: 10.3389/fendo.2023.1170127 (PMC10293789; doi:10.3389/fendo.2023.1170127)
Supplement: Supplementary file 2 [file Table_2.docx]

**Supplementary Table 2 ICD-10 codes for diagnoses**

| **Diagnosis** | **ICD-10** | **n(%)** |
| --- | --- | --- |
| Prediabetes or diabetes | E10/E11/R73 | 108（46.15） |
| Overweight or obesity | E66 | 102（43.59） |
| Hyperlipemia | E78 | 26（11.11） |
| PCOS | E28 | 25（10.68） |
| Hypertension | I10 | 20（8.55） |
| Fatty liver | K76 | 11（4.70） |
| [Hypothyroidism](http://www.baidu.com/link?url=TjUEctb9I2F63tYawUh2PG2zxgzc6kRHW-Lzr4JVns2t9LG8kbkcdPDC0MppWz3ripr4-CDdwxefLtKd07dwNk3DT1JtjDR7NFi5_FDkg9SfwOmSigLHjl_wAhtmhAe6) | E03 | 9（3.85） |
| Metabolic syndrome | E16 | 6（2.56） |
| Acanthosis nigricans | L83 | 4（1.71） |
| Prader-Willi syndrome | Q87 | 2（0.85） |
| Chronic kidney disease | N18 | 2（0.85） |
| Pituitary tumor | E23 | 2（0.85） |
| Hypothalamus syndrome | E23 | 2（0.85） |
